# Current Literature for Veterinarians—Continued

**Published:** 1894-02

**Authors:** 


### EDITORIAL.

### CURRENT LITERATURE.

(Continued from page 54.)

In the last number we gave a list of the strictly veterinary publications which reach us. There are, however, a large number of magazines and papers concerning allied subjects, which are of great advantage to the veterinarian, if not of absolute necessity. These include the journals of human medicine which treat of the advances made in general scientific medicine, and the agricultural, horse and so-called sporting papers, which keep us informed of the market, the results of breeding, and the utility of the animals which we care for.

Wallace's Monthly, monthly, Vol. XIX, published by the American Trotting Register Association, Chicago, Ill. An illustrated magazine devoted to domesticated animal nature. General articles on the horse, more specially treating of the standard-bred trotting horse, and the official register of trotting races.

Spirit of the Times and Sportsman, the American gentleman's newspaper, weekly. Vol. CXXVI, New York. A chronicle of racing, trotting, field sports, aquatics, athletics, and the stage. The official organ of the Board of Control of the American Pony Racing Association and register of running races.

Modern Medicine and the Bacteriological World. Bulletin of the Laboratory of Hygiene, Sanitarium, Battle Creek, Mich. Monthly, Vol. III. An interesting journal, illustrated, containing original articles and extracts upon bacteriological research, germ theory and the treatment of germ diseases, together with general medical subjects.

Johns Hopkins Hospital, Bulletin and Reports, Baltimore, Md. The publications of the Johns Hopkins Hospital, represent a collection of the most advanced and systematic, scientific, medical work done in America. While primarily organized for the human patient, the necessary researches and investigations include comparative medicine to such a degree that they are of equal interest to the veterinarian, and with such names as Osler, Welch, Clement, etc , among the leaders, the veterinarian feels that his profession is rapidly advancing.

THE THERAPEUTIC GAZETTE. A monthly journal of general, special and physiological therapeutics. Vol. XVIII. Philadelphia. A most useful, practical, medical journal, with much of interest to the veterinarian, suggesting many remedies which might be tried on animals with advantage.

THE MEDICAL RECORD. A weekly journal of Medicine and Surgery, Vol. XLIII, New York. Valuable original articles, progress of medical science, editorials, news of the week, society reports, etc., with much that is of practical interest to the veterinarian.

THE NORTHWESTERN LANCET, a semi-monthly medical journal, Vol. XIV, St. Paul, Minn. A live, practical, medical publication.

THE AMERICAN MEDICO SURGICAL BULLETIN, a semi monthly journal of Practice and Science, Vol. VII, New York. A general medical journal. Reports of the New York Academy of Medicine.

THE MEDICAL REVIEW, a weekly Journal of Medicine and Surgery, Vol. XXIX, St. Louis, Mo. A general medical journal, practical, and treats of meat inspection and matters of public health.

THE NEW YORK MEDICAL JOURNAL, a weekly Review of Medicine, Vol. LIX, New York. Lectures and addresses, original communications, leading articles, minor items, society proceedings, etc. A most useful auxiliary for scientific and practical information to the veterinarian's library.

THE NEW YORK MEDICAL EXAMINER, monthly, Vol. IV, New York. The Journal of Insurance Examiners.

ANNALES ET BULLETIN DE LA SOCIÉTÉ DE MÉDECINE DE GAND, Vol. LXXII, Gand, Belgium. A valuable journal of the scientific work of Belgium.

LE PROGRES MÉDICAL, Journal de Médecine, de Chirurgie et de Pharmacie, Vol. XIX, Paris. Weekly. An interesting and valuable practical French Medical Journal.

AGRICULTURAL JOURNAL, published by the Department of Agriculture of the Cape Colony, Africa. Vol. VII. Fortnightly. A useful journal, treating of diseases of animals as well as of valuable agricultural subjects.

THE JERSEY BULLETIN and DAIRY FARMING.—Our field: The Jersey and Dairy World. Vol. XIII. Chicago, Ill. Devoted specially to the interest of the Jersey cattle.

The Holstein Register. Similar to the preceding, treating of the Holstein and Friesian cattle.

The Horseman. Weekly. The Chicago Horseman Newspaper Co., Chicago, Ill.

The Horse World.—A weekly newspaper published at the queen city of the north, Buffalo, N. Y. Vol. X.

Blacksmith and Wheelwright. Published monthly by M. T. Richardson, New York. Contains articles, illustrated, on shoeing horses, iron work and carriage building.

Epitome of Medicine. Published monthly by G. P. Putnam's Sons, edited by James E. Newcomb, M. D. A monthly retrospect of progress in all branches of medicine and surgery.

Public Health in Minnesota. Charles N. Hewitt, editor. Official publication of the State Board of Health. Published monthly. Red Wing, Minn. Contains the reports of the Health officers of the State ; also translations from foreign commissions, etc.

Weekly Drovers' Journal. Published at the Union Stockyards, Chicago. Harvey L. Goodall, proprietor. Reports the markets on cattle, sheep, swine and horses in the country and in Great Britain, together with interesting reading matter on kindred subjects.

Southern Clinic. A monthly journal of medicine, surgery and new remedies, edited by C. A. Bryce, M. D. Richmond, Va.

Scientific American. Published by O. D. Munn, N. Y. City. Publishes descriptions of all the latest patents, and also scientific papers on various subjects.

Forest and Stream. Weekly. New York. General outdoor sports, with special records of all kinds concerning dogs.

Farmers' Friend. Weekly.

The Breeders' Gazette. A weekly journal of live-stock husbandry. Vol. XXI. Chicago, Ill. An interesting agricultural journal.

Zeitschrift fur Fleisch- und Milchhygiene. Published by Dr. med. Robert Ostertag, Professor an der thierärztlichen Hochschule zu Berlin, Berlin. A journal of animal food and sanitary measures.

